# Outcomes measures in a decade of dementia and mild cognitive impairment trials

**DOI:** 10.1186/s13195-016-0216-8

**Published:** 2016-11-21

**Authors:** Jennifer Kirsty Harrison, Anna H. Noel-Storr, Nele Demeyere, Emma L. Reynish, Terry J. Quinn

**Affiliations:** 1 Alzheimer Scotland Dementia Research Centre, University of Edinburgh, c/o Room S1642, Geriatric Medicine, Royal Infirmary of Edinburgh, 51 Little France Crescent, Edinburgh, EH16 4SB UK; 2 Centre for Cognitive Ageing and Cognitive Epidemiology, University of Edinburgh, Edinburgh, UK; 3 Cochrane Dementia and Cognitive Improvement Group, University of Oxford, Oxford, UK; 4 Department of Experimental Psychology, University of Oxford, Oxford, UK; 5 School of Applied Social Sciences, University of Stirling, Stirling, UK; 6 Institute of Cardiovascular and Medical Sciences, University of Glasgow, Glasgow, UK

**Keywords:** Outcome, Dementia, Measurement, Cognition, Function, Quality of life, Mood, Behaviour, Patient-centred

## Abstract

**Background:**

In a research study, to give a comprehensive evaluation of the impact of interventions, the outcome measures should reflect the lived experience of the condition. In dementia studies, this necessitates the use of outcome measures which capture the range of disease effects, not limited to cognitive functioning. In particular, assessing the functional impact of cognitive impairment is recommended by regulatory authorities, but there is no consensus on the optimal approach for outcome assessment in dementia research. Our aim was to describe the outcome measures used in dementia and mild cognitive impairment (MCI) intervention studies, with particular interest in those evaluating patient-centred outcomes of functional performance and quality of life.

**Methods:**

We performed a focused review of the literature with multiple embedded checks of internal and external validity. We used the Cochrane Dementia and Cognitive Improvement Group’s register of dementia studies, ALOIS. ALOIS was searched to obtain records of all registered dementia and MCI intervention studies over a 10-year period (2004–2014). We included both published and unpublished materials. Outcomes were categorised as cognitive, functional, quality of life, mood, behaviour, global/disease severity and institutionalisation.

**Results:**

From an initial return of 3271 records, we included a total of 805 records, including 676 dementia trial records and 129 MCI trial records. Of these, 78 % (630) originated from peer-reviewed publications and 60 % (487) reported results of pharmacological interventions. Cognitive outcomes were reported in 70 % (563), in contrast with 29 % (237) reporting measures of functional performance and only 13 % (102) reporting quality of life measures. We identified significant heterogeneity in the tools used to capture these outcomes, with frequent use of non-standardised tests.

**Conclusions:**

This focus on cognitive performance questions the extent to which intervention studies for dementia are evaluating outcome measures which are relevant to individual patients and their carers. The heterogeneity in measures, use of bespoke tools and poor descriptions of test strategy all support the need for a more standardised approach to the conduct and reporting of outcomes assessments.

**Electronic supplementary material:**

The online version of this article (doi:10.1186/s13195-016-0216-8) contains supplementary material, which is available to authorized users.

## Background

Contemporary evidence-based medicine is built upon a foundation of robust clinical trials. The field of dementia and cognitive impairment has fewer evidence-based interventions than many other common diseases. In this context, it is essential that dementia trials be based on sound design, conduct and reporting. Assessing an intervention requires some measure of treatment effect. The importance of selecting relevant outcome measures in all clinical trials is recognised [1]. Poor choice of outcome measure can weaken or invalidate the results of an otherwise well-conducted trial.

A plausible outcome for a dementia trial is diagnosis of incident dementia or assessment of dementia severity. Dementia diagnosis is evolving, [2, 3]; however, most classifications require evidence of cognitive deficits which cause impairment in daily activities and independence [4]. Outcomes other than clinical assessments, such as neuroimaging and tissue biomarkers, have been used to quantify dementia severity; however, the validity of these surrogate measures has been questioned [5], and in certain fields surrogate outcomes have given results that were not confirmed in subsequent trials with clinical outcomes [6].

Many tools are available for quantifying cognition. These tests can range from short screening tests through to detailed multidomain neuropsychological batteries. The authors of a review of neuropsychological tests in dementia described 59 differing assessments that had been used in studies [7]. Even in a relatively niche area such as post-stroke cognitive and mood disorder, there is substantial heterogeneity in assessment, with the authors of a recent review describing almost as many measurement tools as there were trials [8].

A popular dementia trial outcome paradigm describes conversion from a state of mild cognitive impairment (MCI) to dementia. The defining criterion for conversion is decline in functional ability, so assessment of function is crucial [9]. In fact, robust assessment of daily function is important in all dementia studies. Activity limitation and dependency is feared by patients and carers and has substantial economic impact [10]. There is evidence that dependency, rather than cognitive decline, is the more significant predictor of health-related quality of life [8]. Interventions designed to promote independence were identified as the first of ten research priorities by patients, the public and researchers [9]. Therefore, interventions whose efficacy is tested on change in cognitive function may not capture outcomes of greatest relevance for people with dementia and their carers. Again, many tools are available for assessment of function. Describing function can range from assessment of specific task impairments through daily activity to wider societal participation [11].

In studies of established dementia, assessment of treatment response may look at cognitive and physical function or may describe other manifestations of disease, such as behavioural or mood symptoms. Such measures may directly assess the patient or may take collateral information from a suitable informant, such as family members or carers. Progression of dementia involves a complex interplay of cognitive, physical, behavioural and carer factors. In an attempt to quantifying this concept, global assessment scales have been described.

Traditional assessments tend to have a biomedical focus and may not adequately capture the lived experience of dementia for the individual patient. There is growing international recognition of the need for outcome measures in dementia studies to assess domains beyond simple impairment measures [12]. The U.S. Food and Drug Administration (FDA) advocate that clinical trials in dementia should use a co-primary outcome measure, incorporating cognitive and functional or global assessment measures in drug trials [13]. Patient-reported outcome measures are increasingly recommended, and tools for describing generic and health-related quality of life in dementia are available [14].

Thus, a variety of approaches to performing outcome assessment in dementia trials are available, including cognitive, functional, behavioural and quality of life measures. We aimed to describe the outcome measures used in intervention studies for dementia and MCI. We were particularly interested in the use of functional and quality of life measures because these seem to be the factors of greatest importance to those affected by dementia.

## Methods

We conducted a focused search of contemporary dementia trials, following a methodology that has been used previously to describe outcomes in other disease areas [8, 15]. Although not a systematic review, where applicable, we followed best practice in conduct and reporting as described in Preferred Reporting Items for Systematic Reviews and Meta-Analyses (PRISMA) guidance [16]. A study protocol was pre-specified and registered with the Research Registry resource [17] (reviewregistry78).

### Data source

Our primary data source was the Cochrane Dementia and Cognitive Improvement Group’s (CDCIG’s) register of all dementia trials, ALOIS [18]. ALOIS is a freely accessible electronic database whose aim is to collate information on all trials with a dementia or cognition focus. ALOIS is updated on a continuous basis with trials identified from monthly searches across multiple databases (including MEDLINE, Embase, PsycInfo, Cinahl and Lilacs, in addition to international trial registries, pharmaceutical registries and grey literature sources) [19]. A core team of experienced CDCIG information scientists, supplemented by volunteer support, screen monthly search results and identify relevant trials. Trial characteristics are manually extracted from full-text publications to an electronic template following a structured framework devised by the CDCIG to create consistent annotations across the data set. Trials are assigned a primary ‘study aim’ label, such as “Treatment, Dementia”, or “Treatment, MCI”. The meta-data extracted from each trial within ALOIS is categorised using the PICOTS system, where ‘P’ is characteristics about the trial population; ‘I’ is characteristics about the intervention(s); ‘C’ is characteristics about the comparators; ‘O’ is characteristics about the outcomes measured and the instruments used; ‘T’ is characteristics about the timing and duration of the trial; and ‘S’ is further details about the study design used, such as whether it was double-blind or single-blind. Data are presented as a mix of categorised responses and free text fields, and outcome data are free text with description of outcomes taken verbatim from source.

### Search strategy

A single researcher experienced in systematic review (JKH) performed a two-stage search, first identifying relevant studies for inclusion using the filters within ALOIS and collating study IDs; in the second stage, records were accessed and eligibility was assessed, and all ineligible studies were reviewed by a second reviewer (TJQ) to confirm. Any disagreement was resolved by discussion. We searched ALOIS to identify all dementia and MCI trials published, presented at conferences or registered in a clinical trials database during the period between January 2004 and December 2014 inclusive. We used search filters to limit results to intervention studies, specifically excluding diagnostic accuracy studies. We further filtered results on the basis of primary classification within ALOIS, restricting our search to those studies labelled ‘treatment, dementia’ or ‘treatment, MCI’ and excluding those classified as ‘cognitive enhancement, primary prevention, caregiver focused and other’.

We further refined the list of titles produced by ALOIS, limiting it to studies of human subjects and to those published within our date range. Where a trial data set had more than one ALOIS entry, we favoured the primary publication and excluded duplicates. We classified included trials as pertaining to dementia (where the trial population had a diagnosis of a dementia syndrome, including any pathological sub-classification) and MCI (where the trial population had a diagnosis of MCI or any synonyms describing a state of cognitive impairment that does not fulfil diagnostic criteria for dementia). We excluded studies which were ongoing, in planned/protocol stage, stopped early or whose status was classified as ‘unclear’. Unpublished data were included if sufficient information existed to merit an ALOIS entry graded as ‘study complete’.

### Data collection

We extracted data from ALOIS, supplemented by access to source journal where required, to a pre-specified electronic pro forma. We described data source, year of publication, country, nature of intervention, population studied and outcomes assessed. We purposively did not assess methodology or reporting quality of studies. Because our focus was outcomes as described in published materials, when data were unclear, we did not contact authors of articles or national clinical trial (NCT) registrations for clarification. Where outcome data were not listed on ALOIS or the record appeared incomplete, the original article or NCT link was accessed, if available, to obtain outcome measures.

We categorised data source by journal type. We pre-specified a journal classification based on the journal’s primary focus and grouped them into specialist subject areas using the following labels: complementary & alternative medicine, general medicine, geriatric medicine & gerontology, neurology & neuroscience, nursing, nutrition, old age psychiatry & dementia, pharmacology, psychiatry, psychology and rehabilitation, and ‘other’ where none of these labels were applicable. Categorisation was assessed independently by two authors (JKH and ELR) with involvement of a third (TJQ) in the event of disagreement.

We categorised outcomes using the following labels: cognitive function, activities of daily living (ADL) or functional performance, quality of life, mood, behaviour, global/disease severity measures and institutionalisation (binary). For each of these categories, we included recognised assessments tools or scales, such as the Mini Mental State Examination (MMSE) [20] for cognition or the Barthel Index [21] for ADLs or where authors reported using measures of cognition, function, mood and so forth. We further classified the cognitive tests on the basis of whether the assessment, as described, was a recognised, validated cognitive test; not a recognised test, but extrapolation of the cognitive construct being tested was possible; and a category of ‘unclear’ where the nature of testing was uncertain on the basis of information given. Outcomes were classified independently by two authors (JKH and TJQ) with further review by an expert neuropsychologist (ND).

### Analysis

We described the search process using a PRISMA style flow diagram. We assessed aggregate data for included studies, using simple descriptive statistics. We described number and proportion of total for each category of outcome and tabulated the five most common outcomes for each category. We assessed for temporal trends in outcome assessment, number of trials in each outcome category as a proportion of the total number of trials included from that year, and assessed with the chi-square test for trend. With a similar approach, we assessed outcome used by category of data source. As assessment of function is particularly important for trials in an MCI population, we compared proportions with/without a measure in function in MCI and dementia trials. As a post hoc analysis, we described cognitive and functional outcomes, comparing published and unpublished materials.

## Results

### Search

**Fig. 1:**
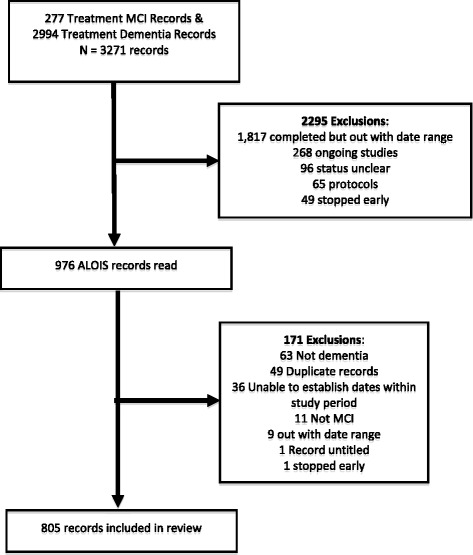
Record selection procedure. *MCI* Mild cognitive impairment

We ran our primary search on 15 May 2015. A total of 3271 records were returned in ALOIS, of which 976 were considered eligible after the first stage. When records were accessed and reviewed in the second stage, 171 were identified by both reviewers as ineligible and were excluded. After filtering and classification, we included a total of 805 records in the review, including 676 dementia trial records and 129 MCI trial records (Fig. 1).

### Study characteristics

The majority of included records were from peer-reviewed publications; however, a significant proportion came from unpublished records and conference abstracts. Pharmacological interventions predominated, making 60 % of those included. Europe and North America were the regions where most included studies were conducted. Individuals with Alzheimer’s disease were the commonest included study population. Most publications were in old age psychiatry & dementia, neurology & neuroscience, and geriatric medicine & gerontology journals (Table 1).

**Table 1:** Characteristics of included records

	*n* (%)
Source	
Peer-reviewed publications	630 (78)
Unpublished	128 (16)
Conference abstracts	47 (6)
Intervention	
Pharmacological	487 (60)
Non-pharmacological	311 (39)
Both	7 (1)
Registration/publication	
2004	40 (5)
2005	54 (7)
2006	68 (8)
2007	79 (10)
2008	99 (12)
2009	125 (16)
2010	68 (8)
2011	96 (12)
2012	59 (7)
2013	80 (10)
2014	33 (4)
2015^a^	4 (0.5)
Region	
Asia	133 (17)
Australasia	27 (3)
Europe	249 (31)
International^b^	46 (6)
Middle East	11 (1)
North America	231 (29)
Russia	13 (2)
South America	16 (2)
Unclear	79 (10)
Dementia subtype	
Alzheimer’s disease	398 (49)
Dementia (unspecified)	158 (20)
Dementia and mild cognitive impairment	30 (4)
Frontotemporal dementia	11 (1)
Mild cognitive impairment	123 (15)
Mixed^c^	39 (5)
Parkinson’s disease dementia/dementia with Lewy bodies	22 (3)
Vascular dementia	24 (3)
Journal of publication (*n* = 630)	
Old age psychiatry & dementia	195 (31)
Geriatric medicine & gerontology	110 (17)
Neurology & neuroscience	95 (15)
General medicine	47 (7)
Pharmacology	44 (7)
Complementary & alternative medicine	27 (4)
Psychiatry	22 (3)
Rehabilitation	20 (3)
Nursing	18 (3)
Nutrition	9 (1)
Psychology	8 (1)
Other	35 (6)

All percentages are rounded to nearest whole number

^a^Denotes study published in 2015 but registered in period of interest

^b^International defined as study sites in more than two regions

^c^Study included those with more than one specific dementia subtype

### Analyses

The frequency of use of each outcome measure and the five commonest measures used for each domain are reported in Tables 2 and 3. Non-reporting of the tool used was common: cognitive (3 %), ADL (34 %), quality of life (23 %), mood (6 %), behaviour (10 %), disease severity and global performance (4 %). Only 49 % of the cognitive measures used were considered recognised cognitive tests. Extrapolation of methods was possible in 36 % of cases, and the remaining 15 % were considered unclear.

**Table 2:** Cognitive and functional outcome measures across included records

	Cognitive		ADL/functional	
Number of records reported^a^	563 (70 %)		237 (29 %)	
Total number of assessments^b^	1278		265	
Number of unique assessments^c^	321		40	
	Assessment tool	*n*	Assessment tool	*n*
	1. Mini Mental State Examination	314	1. No tool	80
	2. Alzheimer’s Disease Assessment Scale – Cognitive Subscale	181	2. Alzheimer Disease Cooperative Study Activities of Daily Living scale^d^	67
	3. Trail Making Tests	45	3. Barthel Index	24
	4. No tool	40	3. Disability Assessment for Dementia	24
	5. Severe impairment battery	27	5. Functional Assessment Staging	7

^a^Number of records in ALOIS where a measure was reported at least one

^b^Total number of times an outcome measure is used in all included records

^c^Total number of unique assessment measures used at least once

^d^Does not include variants of Alzheimer Disease Cooperative Study Activities of Daily Living tool

**Table 3:** Use of other outcome measures across included records

	Quality of life		Mood		Behaviour		Global	
Number of records reported^a^	102 (13 %)		174 (22 %)		303 (38 %)		247 (31 %)	
Total number of assessments^b^	118		207		365		279	
Number of unique assessments^c^	21		41		32		25	
	Assessment tool	*n*	Assessment tool	*n*	Assessment tool	*n*	Assessment tool	*n*
	1. Quality of Life in Alzheimer’s Disease	36	1. Geriatric Depression Scale	47	1. Neuropsychiatric Inventory	183	1. Clinician’s Interview-Based Impression of Change plus caregiver interview	41
	2. No tool	27	2. Cornell Scale for Depression in Dementia	41	2. Cohen-Mansfield Agitation Inventory	53	2. Clinical Dementia Rating – Sum of Boxes	30
	3. EQ-5D	15	3. Brief Psychiatric Rating Scale	13	3. No tool	37	3. Clinical Global Impression	29
	4. DEMQOL/DEMQOL proxy	10	3. Hamilton Rating Scale for Depression	13	4. Neuropsychiatric Inventory – Nursing Home	16	4. Clinical Dementia Rating	28
	5. QUALID and QUALIDEM	4	5. No tool	12	4. BEHAVE-AD	16	5. Clinical Global Impression of Change	27

*Abbreviations: EQ-5D* EuroQol questionnaire, *DEMQOL* Dementia Quality of Life measure, *QUALID* Quality of life in late-stage dementia, *QUALIDEM* a dementia specific quality of life questionnaire rated by professionals, *BEHAVE-AD* Behavioral Pathology in Alzheimer’s Disease

^a^Number of records in ALOIS where a measure was reported at least one

^b^Total number of times an outcome measure is used in all included records

^c^Total number of unique assessment measures used at least once

**Fig. 2:**
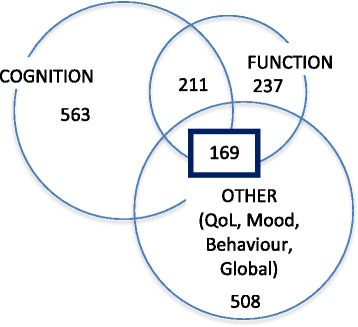
Diagrammatic representation of overlaps in the reporting of cognitive, functional and ‘any other’ outcome measure. *QoL* Quality of life

When published and unpublished studies were considered separately, there was evidence of an association between publication status and the reporting of cognitive outcomes. Cognitive outcomes were reported in 72 % of published studies and 64 % of unpublished studies (*p* = 0.04). There was no evidence of an association between publication status and the reporting of ADL outcomes, which were reported by 30 % of published studies and 27 % of unpublished studies (*p* 0.44). Only 19 (2 %) of studies evaluated institutionalisation as an outcome measure. A complete list of all outcome measures reported is included in Additional file 1. Only 211 (26 %) records included measures of both cognitive performance and functional status; only 169 (21 %) records included measures of cognition and function and any other outcome measure (Fig. 2).

The results of comparative analyses of categorical data are presented in Table 4. There was evidence of greater reporting of mood and quality of life measures in more contemporary trials. There was no evidence of association between time and reporting of the other outcome categories. There was evidence of association between the reporting of mood and behavioural outcomes and publication status, not seen when evaluating the other outcome categories.

**Table 4:** Chi-square analyses of reporting by year and publication status

	Year^a^ (*n* = 801)		Published (yes/no) (*n* = 805)	
	χ^2^ test for trend	*p* value	χ^2^ test	*p* value
Cognition	2.912	0.088	3.750	0.053
Function	1.227	0.268	0.436	0.509
Quality of life	22.693	0.000	1.766	0.184
Mood	12.158	0.000	8.238	0.004
Behaviour	0.226	0.634	11.076	0.001
Global	0.793	0.373	0.757	0.384
Institutionalisation	0.454	0.500	0.240	0.625

^a^Excluded 2015 as not part of study period

Functional measures were more likely to be used in dementia records (217 [32 %] of 676) than in MCI records (20 [16 %] of 129) (proportional difference 16 %, 95 % CI 7.4–24.6 %).

## Discussion

In our evaluation of outcome measures from a decade of contemporary dementia trials, we found substantial heterogeneity in assessment, poor descriptions of assessment tools and a reliance on cognitive measures. There was evidence of greater reporting of mood and quality of life measures in more contemporary trials, but no other trends of improved reporting. Less than one-third included a measure of functional performance, which is needed to establish if the intervention to improve cognitive performance has any practical impact to the individual, and this result was not driven by the inclusion of unpublished material.

Functional measures appeared to be underused in MCI studies, with only 16 % containing any measure of functional/ADL performance. Cognitive function can be considered as an ‘impairment’ measure, summarising where deficiencies in performance are found. In contrast, quality of life and ADL measures focus on what individuals feel or are able to do, and these may be of greater relevance to patients. In particular, when decisions are made about funding of treatments, it is more useful to evaluate the impact of treatment on an individual, rather than the individual’s performance in cognitive testing. It is often unclear how performance on standardized cognitive tests translates into practical or functional outcomes. In the parallel field of stroke, functional assessment is mandatory in stroke trials approved by the FDA, recognising the role of functional recovery after stroke. One difficulty with ADL measures is the presence of ceiling effects [22], and these may be encountered when applied to a population with MCI. However, if the outcome of interest is the conversion from MCI to dementia, assessment of ADL performance is fundamental. MCI trials arguably should all use measures of functional performance to assess the impact of the cognitive impairment on the individual and how the treatment under study affects it.

Only 2 % evaluated the effect of their intervention on institutionalisation. Although institutionalisation in individuals with dementia is likely to be multifactorial [23], it is nonetheless a relevant measure for this population and an outcome feared by those with dementia. There may be scope to use institutionalisation as an outcome, particularly in large, multicentre trials. Determining this outcome may require lengthy longitudinal follow-up if studies target those with early-stage disease.

Of particular concern is that, across all outcome categories, we found a heterogeneity of measures being used. There were also widespread uses of bespoke or unspecified assessments. This not only is problematic for comparisons between studies but also seems unjustified, given the extent of validated tools in each outcome domain. This topic has attracted international attention, with many collaborations attempting to standardise outcome reporting. The International Consortium for Health Outcomes Measurement (ICHOM) initiative has sought to develop outcome sets for specific conditions to standardise the measurement and reporting of outcomes and allow benchmarking in clinical practice, including for dementia [24]. The Core Outcome Measures in Effectiveness Trials initiative has on ongoing project to develop a core outcome set for dementia, although its interest is specifically in the community [25]. The National Institutes of Health Toolbox contains a cognition battery with tests for six domains of cognitive functioning [26]. Taking ICHOM as an example, they recommend use of the following for dementia: the Neuropsychiatric Inventory (NPI), the Montreal Cognitive Assessment, the Bristol Activity of Daily Living Scale, Quality of Life in Alzheimer’s Disease (QOL-AD), Quality of Well-Being Scale – Self-Administered, EuroQol (EQ-5D) and Clinical Dementia Rating [27]. While the QOL-AD and the NPI were the commonest measures of quality of life and behaviour in the trials included in our study, use of the other measures was limited. These recommendations are not universally accepted. All of these calls for standardisation have vocal supporters and opponents, but, to date, few have gained clinical traction, and use of these more standardised approaches has yet to change research or clinical practice. One proposed advantage in the standardisation of outcome assessment is to help facilitate pooling of results and meta-analyses to identify the overall value, or lack thereof, of an intervention. Use of different measures makes this more difficult and can lead to the results obtained from studies using different outcomes being precluded and their data wasted [28]. A potential disadvantage may be that the proposed standard measures are not sufficiently sensitive to pick up small changes in specific cognitive domains, particularly relevant in those with very mild symptoms who are a common target in clinical trials, introducing inefficiencies into research design. Alternatively, any standardised approach represents an opportunity cost to the researchers and patients involved if other measures may be more appropriate.

Although we have highlighted the most commonly used outcome measures, these are not necessarily the most appropriate for dementia studies. There will never be an ideal outcome measure that meets all the requirements of various dementia studies. However, many of the traditionally popular outcome assessments (e.g., MMSE, Barthel Index) were never designed as study outcome measures, and their psychometric properties have been poorly or simply not described. Where many test options are available, choice of the test with best accuracy, reliability and so forth seems intuitive. We note that more contemporary cognitive assessments, including the Oxford Cognitive Screen [29] and Addenbrooke’s Cognitive Assessment III [30], have robust supporting psychometric data. In validation work, these newer measures have been shown to correlate well with functional outcomes. This is encouraging but does not remove the need for separate measures of functional ability.

Cognitive measures were the most varied of those studies, with 321 separate measures used. Just under half of these were considered as accepted cognitive tests. One particular issue was the failure to report the test battery from which measures were derived, instead listing only the test itself, a common reason for a measure to be classed as ‘extrapolation possible’. Best practice in reporting would provide an explicit description of the nature of the test and method used, to allow reproducibility of approach and to reference a source which describes the validity of the tool for the population of interest,

The issues identified are not unique to the field of dementia research. The need for improved reporting of outcomes important to patients and the heterogeneity of used measures has been noted in stroke research [8, 15], and there is a growing recognition of the value of patient-reported outcome measures when evaluating clinical practice [31].

### Strengths and weaknesses

We selected the most recent complete 10-year period to evaluate, providing a contemporary sample of practice. Our study period allows for a description of temporal trends across a decade. Use of ALOIS as a data source brings a breadth of included materials (original research publications, protocols, trial registrations, grey literature) which would not be captured if we restricted our search to electronic databases such as PubMed or Embase. There is attrition between clinical trial registration and subsequent publication [32]. There is also known to be a significant delay in the publication of clinical trials from abstract stage and that many abstracts remain unpublished [33]. Although data derived from an unpublished record may be less comprehensive than those in a peer-reviewed publication, they indicate the a priori intentions of the researchers in the design of their study and the outcome measures deemed important. ALOIS also facilitates the inclusion of foreign language publications because it draws on the resources of the Cochrane Dementia Group and text can be translated, again broadening representation of the research field. Finally, our methods of checking information within the team and accessing specialist expertise help to provide external validity to our findings.

However, we acknowledge that the primary purpose of ALOIS is not about the ascertainment of outcome measures, but rather to register clinical studies in the field of dementia. As such, it is possible that not all outcomes will have been included, and there may be errors in their recording. We supplemented the use of ALOIS where the outcomes list appeared to have been truncated, locating clinical trial registration data or full-text articles where available. We limited our analysis to the data as presented and did not contact authors for clarification. We downgraded materials where ambiguity was found, and this may overestimate the problems of reporting if validated measures were used but not recorded in ALOIS. There is also potential for the misclassification of studies within ALOIS because records can be given only one category. We were interested in common forms of dementia and thus excluded records specific to highly specialised presentations (e.g., Huntington disease, Down syndrome). We also found records which were not specific to a population with MCI or dementia (e.g., older adults, care home residents), which we excluded from our analyses. Our focus was on describing outcomes, and we did not assess the quality of design, conduct or reporting of the included studies. It would be of interest to assess whether overall study quality was associated with use of well-described, multimodal outcome assessment.

### Suggestions for future trials

The choice of outcome measures for an intervention study will vary, depending on the population, study design and methods. Given the range of challenges experienced by individuals with dementia and the complexity of the domains affected by the causative conditions within the umbrella of dementia, no single group of measures will be appropriate for every study. We advocate greater use of outcome measures which capture the effect of the intervention on the lived experience of the individual with dementia or MCI. Trialists should ensure outcome measures, including test batteries, are fully described and make greater use of validated, standardised measures. We encourage researchers to describe not only which tests were used, with supporting references, but also how these tests were employed in practice. It is also vital that, for any outcome instrument, we understand the feasibility of use, reliability and other test properties so that results can be more readily applied to clinical practice. Reporting guidance, such as the Consolidated Standards of Reporting Trials (CONSORT) statement [34], has a key role in ensuring that researchers are explicit in describing reproducible methods and using validated assessment.

## Conclusions

In this review, we sought to quantify the breadth of reporting of outcome measures in dementia research. Having identified the dominance of cognitive measures and lack of use of measure of ADL performance, these issues require urgent attention. We also identified problems of unclear reporting and heterogeneity of measures, which increase the potential for research waste. These require a more standardised approach to be adopted within the field which has greater potential for patient benefit as results can be harmonised and compared.

## Additional file

Additional file 1: Full list of outcome measures reported. Tables including all outcome measures reported in each of the categories: cognitive, functional/ADL, quality of life, mood, behaviour and global. (DOCX 37 kb)

## Abbreviations

ADCS-ADL: Alzheimer’s Disease Cooperative Study Activities of Daily Living Inventory
ADL: Activities of daily living
BEHAVE-AD: Behavioral Pathology in Alzheimer’s Disease
CDCIG: Cochrane Dementia and Cognitive Improvement Group
CMAI: Cohen-Mansfield Agitation Inventory
CONSORT: Consolidated Standards of Reporting Trials
DEMQOL: Dementia Quality of Life measure
EQ-5D: EuroQol questionnaire
FDA: U.S. Food and Drug Administration
ICHOM: International Consortium for Health Outcomes Measurement
MCI: Mild cognitive impairment
MMSE: Mini Mental State Examination
NCT: National clinical trial
NPI: Neuropsychiatric Inventory
PRISMA: Preferred Reporting Items for Systematic Reviews and Meta-Analyses
QOL-AD: Quality of Life in Alzheimer’s Disease
QUALID: Quality of life in late-stage dementia
QUALIDEM: a dementia specific QOL questionnaire rated by professionals
