# Supplementary material for: Outcomes measures in a decade of dementia and mild cognitive impairment trials
Source: Alzheimers Res Ther. 2016 Nov 21;8:48. doi: 10.1186/s13195-016-0216-8 (PMC5116815; doi:10.1186/s13195-016-0216-8)
Supplement: Additional file 1: — Full list of outcome measures reported. Tables including all outcome measures reported in each of the categories: cognitive, functional/ADL, quality of life, mood, behaviour and global. (DOCX 37 kb) [file 13195_2016_216_MOESM1_ESM.docx]

Additional file 1: Full list of outcome measures reported

Cognitive measures – 563 records (70%) on 1278 occasions; 321 different measures used

| **COGNITIVE TEST** | **EXTRAPOLATION POSSIBLE** | **UNCLEAR** |
| --- | --- | --- |
| Aachener aphasia test | 5-minute delayed recall scores | Cognition – no tool |
| Abbreviated Mental Test (AMT) |  |  |
| Addenbrookes Cognitive Assessment – Revised (ACE-R) | 8 words test | Memory – no tool |
| Alzheimer’s Disease Assessment Scale cognitive subscale (ADAS-cog) | Animal fluency | Age task |
| Age-adjusted concentration test (AKT) | Animal naming | Assessment of motor and process skills |
| Alzheimer's Quick test | Bizareness | Attention |
| Amsterdam Dementia Screening Test 6 | Calculation ability | Attention and Concentration |
| Attention Network Test | Changes in neuropsychological and functional behaviours as observed by the primary caregivers and the subject | Attention task |
| Attentive matrices | Choice reaction time | Attentional performance |
| Auditory emotion recognition task | Clock recognition | Changes in thinking and memory |
| Auditory verbal learning test | Color word test | Cognitive flexibility |
| Auditory-Verbal Task | Complex visual scene encoding task (CVSET) | Cognitive Performance Change |
| Autobiographical memory interview | Comprehension | Cognitive test battery |
| Backward counting and walking (dual task) | Computerized dot counting test | Colour task |
| Backward Digit Span | Computerized stroop test | Computerized neuropsychological test battery |
| Barrow Neurological Institute Screen for Higher Cerebral Functions (BNIS) | Constructional praxis | Concentration/distractability |
| Battery for analysis of aphasic deficits | Constructional recall | Continuity of attention |
| Behavioural Assessment of the Dysexecutive Syndrome (BADS) | Controlled oral word association test | Direction memory |
| Benton orientation test | Copy of simple drawings | Drawing |
| Benton visual retention test form F&G |  |  |
| Block Design | Delayed cue recall | Errors |
| Boston Naming Test | Delayed pictures recall | Examination of memory and temporality |
| Brief Assessment of Prospective memory short form | Delayed recall of short stories | Executive function |
| Brief cognitive rating scale | Delayed recall subscale | Executive Interview |
| Brief cognitive screen battery | Delayed story recall score | First item |
| Brief test of attention | Delayed word recall task | fMRI cognitive task |
| Brief visuospatial memory test | Digit span | Group differences in cognitive performance |
| Buschke Selective Reminding Rask | Digit symbol coding | Language |
| California Verbal Learning Test | Dual task performance | Language comprehension |
| Cambridge Cognitive Examination (CAMCOG) | Episodic memory | Learning |
| CANTAB | Event-related prospective memory task | Memory clinic protocol |
| Card rotation test | Executive cognitive test of selective attention | Memory function questionnaire |
| Cognitive abilities screening instrument (CASI) | Face and picture recognition | Memory measures |
| Categorization Working Memory Span (CWMS) | Face matching task | Motor task performance |
| Category Fluency | Face-name associations memory tasks | Multifactorial memory questionnaire |
| Category naming test | Facial expression recognition task | Neuropsychological Test battery |
| Category verbal fluency test | Free recall test | Objects |
| Cerebraler insutfizienz test | Go/no go paradigm | Power of attention |
| Changes in the international study of post-operative cognitive decline and the CogHealth computerized battery tests | Immediate recall | Primacy |
| Clinical Memory Scale | Inhibitory control | Psychomotor speed |
| Clinical Dementia Rating (CDR) | Instant memory | Quality of episodic secondary memory |
| Clock drawing test | Judgement of line orientation | Quality of working memory |
| Cognitive Capacity Screening Examination (CCSE) |  |  |
| Cognitive Drug Research (CDR) Battery | Key search test | Recall |
| Cognitive Drug Research (CDR) Computerised Testing | Learning/memory and frontal executive skills | Recency |
| Cognitive drug research test | Letter fluency test | Repetition |
| Cognitive estimation test; | Letter verbal fluency test | Retrospective functioning |
| Cognitive Performance Scale (CPS) | List Learning | Route test |
| CogScreen Test Battery | List recall test | Seriousness of forgetting |
| Cogstate battery - working memory | Location matching task | Short-term memory |
| CogState computerised neurological test battery | Long term verbal memory | Source recognition task |
| Complex figure tests | Long-delay free recall changes | Test Barcelona |
| Composite Cognitive Memory Score | Long-delay recognition | Visuospatial ability |
| Consortium to Establish a Registry for Alzheimer’s Disease (CERAD) | Memorizing face-scene pairs | Working memory |
| Consortium to Establish a Registry for Alzheimer's disease (CERAD) delayed recall test | Memory list learning |  |
| Corsi's test |  |  |
| Delis Kaplan Executive Function System test battery (D-KEFS) | Mental Rotations Task |  |
| DemTect | Mnemonics usage |  |
| Digit cancellation test | Naming |  |
| Digit Symbol Substitution Test (DSST) | n-Back Test |  |
| DMS 48 task | Oral action naming |  |
| Dot Matrix Test | Oral object naming |  |
| Episodic memory RI-48 cued recall test | Orientating ability of time and space |  |
| Everyday problems test | Paragraph recall |  |
| FAS test of verbal fluency | Pattern Comparison Test |  |
| Figural Memory Test | Phonemic Fluency |  |
| Five words test | Picture encoding phase |  |
| Five-Cog | Picture free memory |  |
| French Rapid Evaluation of Cognitive Function | Picture naming performance |  |
| Frontal assessment battery (FAB) | Picture-naming task |  |
| Frontal Behavioural Inventory (FBI) | Proactive interference |  |
| Frontal dysfunction battery | Reaction time |  |
| Fuld Object Memory Evaluation | Reading Span Task |  |
| Global Deterioration Scale (cognition) | Recognition memory |  |
| Graphic recognition test (GRT) | Recognition Memory Test - Faces |  |
| Hasegawa's Dementia Scale | Recognition Training Task |  |
| Hasegawa's Dementia Scale - revised (HDS-R) | Repetition-Lag procedure |  |
| Hong Kong List Learning Test | Selective attention measure |  |
| Hopkins verbal learning test | Selective reminding test |  |
| Hopkins Verbal Learning Test Revised | Semantic fluency |  |
| Letter digit substitution test | Semantic memory |  |
| Letter Number Sequencing (LNS) | Sentence comprehension |  |
| Logical Memory 1 | Serial curve position |  |
| Logical Memory 2 | Short story recall |  |
| Manchester and Oxford Universities Scale for the Psychological Assessment of Dementia (MOUSEPAD) | Short story test |  |
| Matrix reasoning | Short term verbal memory |  |
| Maze task | Short-delay free recall changes |  |
| Maze-A2 | Simple Drawings |  |
| Measure of attention (CoA) | Simple reaction time |  |
| Memory awareness rating scale (MARS) | Spatial ability measure |  |
| Memory Failures in Everyday Memory | Spatial memory measure |  |
| Memory quotient (MQ) | Spatial recall |  |
| Milan Overall Dementia Assessment Battery | Speed of memory |  |
| Mini Mental State Examination (MMSE) | Speed of processing |  |
| Montreal Cognitive Assessment (MoCA) | Story encoding phase |  |
| New York University NYU Paragraph Delayed Recall Test | Story recall |  |
| Number cancellation test | Trail making test |  |
| Oral word association tests (OWAT) | Trails Set-Shifting task (modified) |  |
| Paced Auditory Serial Addition Task (PASAT) | Verbal ability measure |  |
| Paired associated learning subtests | Verbal digits tests |  |
| PHOTOTEST | Verbal fluency test |  |
| Poppelreuter-Ghent Overlapping Figures Test | Verbal learning |  |
| Psychologix and Cogscreen Test Batteries | Verbal memory |  |
| Psychologix Computerized Cognitive Test | Visual memory span |  |
| Pyramids and Palm Trees Test | Visual memory task |  |
| Raven's coloured matrices | Visual motor integration |  |
| Repeatable battery for assessment of cognitive status (RBANS) | Visual recognition task - attention, working memory, recognition memory |  |
| Repeatable Battery for the Assessment of Neuropsychological States - Japanese version (RBANS) | Visual retention-I test |  |
| Repeatable Battery for the assessment of neuropsychological status (RBANS) | Word fluency |  |
| Revised Hasegawa Dementia Scale | Word fluency tests |  |
| Rey Auditory Verbal Learning Test | Word list |  |
| Rey Kim memory quotient | Word list delayed recall |  |
| Rey-osterrieth complex figure test; | Word list learning tests |  |
| Rey's auditory-verbal learning test | Word list memory |  |
| Rivermead Behavioural Memory Test | Word list recall |  |
| RL/RI 16 free and cued recall task | Word list recognition |  |
| Self-Ordered Pointing test | Word list savings score |  |
| Seoul Neuropsychological Screening Battery | Word list total immediate recall |  |
| Seoul Neuropsychological Screening Battery - Dementia version | Word recall |  |
| Severe impairment battery (SIB) | Word recognition |  |
| Sheffield Test for Acquired Language Disorders |  |  |
| Shipley inference scale |  |  |
| Shipley vocabulary scale |  |  |
| Short cognitive performance test (SKT) |  |  |
| Signoret's Memory Battery |  |  |
| Similarities and Matrix Reasoning |  |  |
| Single Digit Modality Test |  |  |
| Spot-the-word |  |  |
| Stockholm Gerontology Research Center Test of Memory for Words |  |  |
| Stop signal task |  |  |
| Stroop Test |  |  |
| Subject Global Impression-Cognition (SGI-Cog) scale |  |  |
| Sustained attention to response task |  |  |
| Symbol digit modalities test |  |  |
| Syndrom Kurz test |  |  |
| Telephone interview of cognitive status |  |  |
| Test for severe impairment |  |  |
| Test of everyday attention |  |  |
| Token test |  |  |
| Tracking question |  |  |
| Verbal Learning and Memory Test (VLMT): List A - delayed recall and recognition and direct recall |  |  |
| Verbal learning and memory tests by additional cognition composite |  |  |
| Verbal span |  |  |
| Visual paired associates |  |  |
| Waterline task |  |  |
| Wechsler |  |  |
| Wechsler Adult Intelligence Scale |  |  |
| Wechsler Adult Intelligence Scale - revised |  |  |
| Wechsler Adult Intelligence Scale III |  |  |
| Wechsler Memory Scale |  |  |
| Wechsler Memory Scale - revised |  |  |
| Wechsler Scale Subsets |  |  |
| Weigl's sorting test |  |  |
| Western Aphasia Battery |  |  |
| Wide range achievement test |  |  |
| Wisconsin Card Sorting Test |  |  |

Functional – 237 (29%) of records; 265 occasions; 40 measures

| **Tool** | **Number of studies** |
| --- | --- |
| No tool | 80 |
| Activities of daily living questionnaire of Alzheimer disease - Portuguese version | 1 |
| ADCS-ADL (Alzheimer disease cooperative study ADL scale) | 67 |
| ADCS-ADL MCI | 1 |
| ADCS-IADL (Alzheimer disease cooperative study IADL scale) | 2 |
| ADL-IS (international scale) | 2 |
| Alzheimer's Disease Functional Assessment and Change Scale | 1 |
| Barthel index | 24 |
| BAYER activities of daily living scale | 2 |
| Bedford Alzheimer Nursing Severity Scale (BANSS) | 1 |
| Blessed Functional Activity Scale | 1 |
| Bristol Activities of Daily Living Scale (BADLS) | 4 |
| Canadian Occupational Performance Measure | 2 |
| Caregiver reported ADL performance | 1 |
| Direct Assessment of ADL performance | 1 |
| Direct Assessment of Functional State - revised (DAFS-BR) | 1 |
| Direct Assessment of Functional Status (DAFS) | 2 |
| Disability Assessment for Dementia (DAD) | 24 |
| Erlangen Test of ADL (E-ADL) | 2 |
| Everyday Cognition (E-cog) | 1 |
| Functional Activities Questionnaire | 2 |
| Functional assessment and change scale | 1 |
| Functional Assessment staging (FAST) | 7 |
| Functional independence measure | 4 |
| Functional Rating Scale for Symptoms of Dementia | 2 |
| Groningen Activity Restriction Scale | 1 |
| Interview for Deterioration of Daily Activities in Dementia | 6 |
| Katz index | 1 |
| Korean Instrumental Activities of Daily Living | 4 |
| Lawton scale | 4 |
| Level of activity by Tokyo metropolitan institute of gerontology index life space assessment | 1 |
| MDS-ADL | 1 |
| Nurse informant ADL scale | 1 |
| Performance-based assessment of instrumental activities of daily living (PASS) | 1 |
| Pfeffer Functional Activities Questionnaire | 2 |
| Physical Self Maintenance Scale (PSMS) | 2 |
| Psycho-geriatric dependency rating scale | 1 |
| Schwab and England scales | 1 |
| Seven-item minimum data set activities of daily living MDS-ADL | 1 |
| Standardised 20-item ADL scale | 1 |
| Texas Functional Living scale | 1 |

Quality of Life – 102 records (13%); 118 uses; 21 measures used

| **Tool** | **Number of studies** |
| --- | --- |
| No tool | 27 |
| Bath Assessment of Subjective Quality of Life in Dementia (BASQID) | 1 |
| Blau QOL scale | 1 |
| Cornell-Brown QOL | 1 |
| Dementia Quality of Life Score | 2 |
| DEMQOL/DEMQOL Proxy | 10 |
| EQ-5D | 15 |
| GHQ | 2 |
| Medical Outcomes 36 item Short Form Health Survey | 2 |
| NPI-Q | 1 |
| PD QoL measures | 1 |
| Perceived Deficits Questionnaire (PDQ) | 1 |
| Personal well-being index | 1 |
| Personal wellbeing index for intellectually disabled (PWI-ID) | 1 |
| PHQ | 1 |
| QoL-AD | 36 |
| QUALID | 4 |
| QUALIDEM | 4 |
| SF-12 | 3 |
| SF-36 | 2 |
| SF-8 | 1 |
| Smiley Face Rating Scale (wellbeing) | 1 |

Mood – 174 studies (22%); 207 uses of 41 measures

| **Tool** | **Number of studies** |
| --- | --- |
| No tool | 12 |
| Alzheimer's Mood Scale (AMS) | 3 |
| Apparent affect rating scale | 2 |
| BASDEC depression scale; | 1 |
| Beck anxiety inventory | 2 |
| Beck Depression Inventory (BDI) | 6 |
| Beck Inventory | 1 |
| Bond-Lader Visual Analogue Scales | 1 |
| Brief Psychiatric Rating Scale | 13 |
| Center for Epidemiology Studies Depression Scale (CES-D) | 12 |
| CERAD (for depression) | 2 |
| Cornell Scale for Depression in Dementia | 41 |
| Dementia Apathy Interview Rating (DAIR) | 1 |
| Dementia Mood Assessment Scale (DMAS) | 2 |
| Dementia Mood Picture Test; | 1 |
| Depression Anxiety Stress Scale | 2 |
| Depression rating scale of the minimum data set of the RAI | 1 |
| Direct observation | 1 |
| FACE Facial expression Scoring | 2 |
| General Health Questionnaire | 2 |
| Geriatric Anxiety Inventory | 1 |
| Geriatric Depression Scale (GDS) | 47 |
| Hamilton Depression Rating Scale (HDRS) | 13 |
| Hamilton Scale for Anxiety | 1 |
| Hospital Anxiety and Depression Scale (HADS) | 5 |
| Mental status in neurology MS | 1 |
| Montgomery Asbery Depression Scale (MADRS) | 4 |
| NPI - mood domains | 1 |
| Observed affect scale | 1 |
| Observed emotion rating scale | 2 |
| Olin criteria | 1 |
| Philadelphia Geriatric Center Affect Rating Scale | 4 |
| Philadelphia Geriatric Center Morale Scale | 2 |
| PHQ-9 | 2 |
| Positive and negative affect scale | 1 |
| Profile of Mood States | 1 |
| Raskin depression scale | 1 |
| Rating Anxiety in Dementia | 7 |
| SCL-90 (symptom checklist); | 1 |
| Stroke Aphasic Depression Questionnaire | 1 |
| Structured clinical interview for DSM-IV Axis I Disorders (SCID-DSM IV) | 1 |
| The Spilberger Scale | 1 |

Behaviour 303 (38%) of studies; 365 occasions; 32 measures used

| **Tool** | **Number of studies** |
| --- | --- |
| No tool | 37 |
| Agitated behaviours in dementia scale | 4 |
| Agitation Behaviour Mapping Instrument | 1 |
| Apathy evaluation scale | 7 |
| Apathy Scale for Institutionalized Patients with Dementia | 1 |
| BEHAVE-AD | 14 |
| Behavior Rating Scale for the Elderly (BRSE) | 1 |
| Behaviour Assessment Scale of Later Life (BASOLL) | 1 |
| Behaviour engagement and agitation measure (BEAM) | 1 |
| Behaviour rating inventory of executive function - adult version | 1 |
| Behavioural Assessment of the Dysexecutive Syndrome (BADS) | 2 |
| Beooddelingsschall voor Oudere PaieEnten (BOP) (behaviour evaluation scale) | 1 |
| Blessed behaviour measuring scale | 2 |
| Brief Agitation Rating Scale (BARS) | 3 |
| Clifton Assessment Procedures for the Elderly Behaviour Rating Scale (CAPE-BRS) | 3 |
| Cohen Mansfield Agitation Inventory (CMAI) | 53 |
| Crichton Royal Behavior Rating Scale | 1 |
| Dementia Behavior Disturbance Scale (DBDS) | 2 |
| Dysfunctional Behaviour Rating Instrument | 1 |
| E-BEHAVE-AD Empirical Behavioural Pathology in AD | 3 |
| Frontal behavioural inventory | 2 |
| Frontal System behaviours scale | 3 |
| INTERACT | 2 |
| Lawton's Modified Behaviour Stream | 1 |
| London Psychogeriatric Rating Scale | 1 |
| Neurobehavioural rating scale | 3 |
| Neuropsychiatric Inventory (NPI) | 183 |
| Neuropsychiatric Inventory (Nursing Home Version) (NPI-NH) | 16 |
| Passivity in Dementia Scale | 1 |
| Pittsburgh Agitation Scale (PAS) | 1 |
| Rating Scale for Aggressive Behaviours in the Elderly (RAGE) | 1 |
| Revised Memory and Behaviour Problems Checklist | 10 |
| Staff Observation Aggression Scale | 2 |

Global/disease progression – 247 (31%) of studies, 279 occasions, 25 measures

| **Tool** | **Number of studies** |
| --- | --- |
| No measure | 11 |
| ADCS-CGIC | 26 |
| ADCS CGIC-MCI | 1 |
| Bedford Alzheimer Nursing Severity Scale | 1 |
| Blessed Dementia Rating Scale | 5 |
| Clinical Dementia Rating (CDR) | 28 |
| Clinical Dementia Rating (CDR) sum of boxes | 30 |
| Clinical Global Impression (CGI) | 29 |
| Clinical Global Impression of Change (CGI-C) | 27 |
| Clinical Global Impression – Improvement (CGI-I) | 3 |
| Clinical Global Impression – Severity (CGI-S) | 11 |
| Clinician's Interview Based Impression of Change (CIBIC) | 10 |
| Clinician's Interview Based Impression of Change plus caregiver input (CIBIC-plus) | 41 |
| Dementia Rating scale | 4 |
| Dementia Rating Scale-2 (DRS-2) | 2 |
| Dementia Severity Rating Scale | 3 |
| Global deterioration scale | 20 |
| Global function by caregiver interview questionnaire | 1 |
| Gottfries-Brine-Steen Scale | 2 |
| Hasegawa Dementia Scale-Revised | 1 |
| Hasegawa's Dementia Scale | 1 |
| Hierarchic dementia scale (HDS) | 1 |
| Mattis Dementia Rating Scale | 12 |
| NOSGER Cumulative Value | 6 |
| Progressive Deterioration Scale | 2 |
| The Patient Global Assessment (PGA) | 1 |
